# Joint contractures in the absence of inflammation may indicate mucopolysaccharidosis

**DOI:** 10.1186/1546-0096-7-18

**Published:** 2009-10-23

**Authors:** Rolando Cimaz, Giovanni Valentino Coppa, Isabelle Koné-Paut, Bianca Link, Gregory M Pastores, Maria Rua Elorduy, Charles Spencer, Carter Thorne, Nico Wulffraat, Bernhard Manger

**Affiliations:** 1 Ospedale Meyer-Reumatologia, Firenze, Italy; 2 Department of Maternal-Infantile Sciences, Polytechnic University of Marche, Ancona, Italy; 3 Department of Pediatrics, Pediatric Rheumatology, CHU de Bicêtre, Le Kremlin Bicêtre, France; 4 Johannes Gutenberg-University, Villa Metabolica, Mainz, Germany; 5 Department of Neurology and Pediatrics, New York University School of Medicine, New York, NY, USA; 6 Division of Metabolism, Paediatric Rheumatology Unit, Cruces Hospital, Barakaldo, Spain; 7 Nationwide Children's Hospital, Columbus, OH, USA; 8 Southlake Regional Health Centre, The Arthritis Program, Newmarket, Ontario, Canada; 9 Department of Pediatric Immunology, University Medical Center Utrecht, Utrecht, Netherlands; 10 Department of Clinical Immunology and Rheumatology, Department of Medicine III, Erlangen Medical School, Erlangen, Germany

## Abstract

**Background:**

Undiagnosed patients with the attenuated form of mucopolysaccharidosis (MPS) type I often have joint symptoms in childhood that prompt referral to a rheumatologist. A survey conducted by Genzyme Corporation of 60 European and Canadian rheumatologists and pediatric rheumatologists demonstrated that < 20% recognized signs and symptoms of MPS I or could identify appropriate diagnosis tests. These results prompted formation of an international working group of rheumatologists, pediatric rheumatologists, and experts on MPS I to formulate a rheumatology-based diagnostic algorithm. The resulting algorithm applies to all MPS disorders with musculoskeletal manifestations.

Bone and joint manifestations are prominent among most patients with MPS disorders. These life-threatening lysosomal storage diseases are caused by deficient activity of specific enzymes involved in the degradation of glycosaminoglycans. Patients with attenuated MPS disease often experience diagnostic delays. Enzyme replacement therapy is now commercially available for MPS I (laronidase), MPS II (idursulfase), and MPS VI (galsulfase).

**Presentation of the hypothesis:**

Evolving joint pain and joint contractures in the absence of inflammation should always raise the suspicion of an MPS disorder. All such patients should undergo urinary glycosaminoglycan (uGAG) analysis (not spot tests for screening) in a reputable laboratory. Elevated uGAG levels and/or an abnormal uGAG pattern confirms an MPS disorder and specific enzyme testing will determine the MPS type. If uGAG analysis is unavailable and the patient exhibits any other common sign or symptom of an MPS disorder, such as corneal clouding, history of hernia surgery, frequent respiratory and/or ear, nose and throat infections; carpal tunnel syndrome, or heart murmur, proceed directly to enzymatic testing. Refer patients with confirmed MPS to a geneticist or metabolic specialist for further evaluation and treatment.

**Testing of the hypothesis:**

We propose that rheumatologists, pediatric rheumatologists, and orthopedists consider our diagnostic algorithm when evaluating patients with joint pain and joint contractures.

**Implications of the hypothesis:**

Children and young adults can suffer for years and sometimes even decades with unrecognized MPS. Rheumatologists may facilitate early diagnosis of MPS based on the presenting signs and symptoms, followed by appropriate testing. Early diagnosis helps ensure prompt and appropriate treatment for these progressive and debilitating diseases.

## Background

Undiagnosed patients with the attenuated form of mucopolysaccharidosis type I (MPS I) often have joint symptoms in childhood that prompt their referral to a rheumatologist, orthopedist, or orthopedic surgeon. Diagnostic delays are unfortunately common for patients with attenuated MPS I, who can suffer for years, sometimes decades, from this progressive and debilitating disease before it is finally recognized [1-4] (Appendix). The advent of enzyme replacement therapy (laronidase, Aldurazyme^®^, Genzyme Corporation, Cambridge MS) for MPS I in 2003, has enabled a targeted approach in the management of attenuated MPS I. Rheumatologists and musculoskeletal specialists are in a position to recognize the possibility of MPS I, enabling appropriate diagnosis and treatment of affected individuals before development of an irreversible disease stage.

To gauge physician familiarity with the symptoms of MPS I and experience with MPS I patients, Genzyme Corporation conducted a survey of practicing rheumatologists and pediatric rheumatologists in Canada, France, Germany, Italy, Spain, and the United Kingdom [4]. Among the 60 participants, only 9% indicated that they had seen an MPS I patient in their practice; however, an additional 13% indicated that they might have an MPS I patient in their care on the basis of common signs and symptoms. When presented with a description of an 8-year-old girl or a 23-year-old woman exhibiting musculoskeletal signs of MPS I, only 13% of pediatric rheumatologists and 19% of rheumatologists considered MPS I in their differential diagnosis. This percentage increased only slightly or actually declined when additional signs and symptoms of MPS I were added to patient descriptors. Finally, when asked what diagnostic tests should be performed when MPS I is suspected, only 20% of the pediatric rheumatologists and none of the rheumatologists identified appropriate tests. The results of this survey prompted an international working group of rheumatologists, pediatric rheumatologists, and experts on MPS I to formulate a rheumatology-based diagnostic algorithm for MPS I.

Although the algorithm was developed specifically for MPS I, it is also applicable to attenuated forms of all other MPS disorders with the exception of MPS III (Sanfilippo syndrome), which is characterized primarily by CNS manifestations [5-7]. In particular, MPS I and II (Hunter disease) have a very similar clinical presentation, although MPS II occurs primarily in boys as it is inherited in an X-linked recessive manner. MPS II and MPS VI (Maroteaux-Lamy) are also now treatable with enzyme replacement therapy [8,9].

### About MPS I

MPS I is one of the more common mucopolysaccharidoses, a family of seven inherited metabolic diseases caused by deficient activity of various lysosomal enzymes, resulting in the inability to metabolize certain glycosaminoglycans (Table 1). In the case of MPS I, patients have an undetectable activity of the lysosomal enzyme α-L-iduronidase (IDUA)[10]. Lack of IDUA activity results in progressive accumulation of the glycosaminoglycans dermatan sulfate and heparan sulfate in lysosomes throughout the body, leading to a multi-systemic disease. Prominently affected organ systems include airways, heart, viscera, skeleton, and (in the most severe cases) the central nervous system. The disease presents across a spectrum of severity, and historically is categorized into three subjectively defined phenotypes. "Hurler syndrome," the most severe form of the disease, begins in infancy, progresses rapidly, and is associated with profound cognitive decline. Untreated children with Hurler syndrome rarely survive beyond age 10 [11]. "Scheie syndrome," the least severe, is characterized by onset in childhood, variable rate of progression and organ involvement, normal intelligence, and survival to adulthood. "Hurler-Scheie syndrome" encompasses patients with onset in early childhood, mild to moderate CNS involvement, and an intermediate rate of progression and organ involvement. Clinical heterogeneity is especially prominent in the less severe end of the spectrum, and Hurler-Scheie and Scheie presentations are often grouped together and referred to as "attenuated MPS I."

**Table 1:** MPS Disorders Likely to be Encountered by a Rheumatologist or Orthopedist

	**MPS I** **(Hurler, Hurler-Scheie, Scheie)**	**MPS II** **(Hunter)**	**MPS IV** **(Morquio)**	**MPS VI** **(Maroteaux-Lamy)**	**MPS VII** **(Sly syndrome)**
**Deficient lysosomal enzyme**	α-L-iduronidase	Iduronate sulfatase	Galactose 6-sulfatase or β-galactosidase	Arylsulfatase B	β-Glucuronidase

**Inheritance**	Autosomal recessive	X-linked recessive (most patients are male)	Autosomal recessive	Autosomal recessive	Autosomal recessive

**Common clinical** **manifestations in** **attenuated patients** **(can be subtle or severe)**					

Joint involvement with no inflammation	+	+	+ *	+	+

Dysostosis multiplex	+	+	+ *	+	+

Growth deficits	+	+	+	+	+

Corneal clouding	+	None	+	+	~

History of umbilical and/or inguinal hernia	+	+	+	+	+

Elevated total urinary glycosaminoglycan level^† ^ and/or abnormal glycosaminoglycan profile	+	+	+^§^	+	+

Coarse facies	+	+	+	+	+

Carpal tunnel syndrome	+	+	~	+	+

Cardiac valve disease	+	+	+	+	+

**Enzyme replacement therapy**	Laronidase (Aldurazyme^®^)	Idursulfase (Elaprase^®^)	In development	Galsulfase (Naglazyme^®^)	In development

*MPS IV is characterized by distinctive and severe skeletal dysplasia, dysplastic odontoid process, and joint hyper-extendibility/ligamentous laxity.

^†^False negatives can occur with spot screening.

^§^Patients with MPS IV typically have an abnormal GAG profile, but total urinary GAG levels can be in the normal range with some methodologies

Patients with attenuated MPS I, unlike those with the severe, "Hurler" form of the disease, often have no obvious physical abnormalities; facial appearance is usually normal or only subtly altered, stature can be in the normal range, and skeletal abnormalities are often absent or subclinical. Learning issues, if present, can be mild. Common early clinical features include joint pain and stiffness, corneal clouding, umbilical and/or inguinal hernia, carpal tunnel syndrome (note that an MPS disorder is the most frequent cause of carpal tunnel syndrome in children and adolescents [12,13]), hearing loss, frequent ear, nose and throat infections, and "noisy" breathing. Musculoskeletal abnormalities that can occur later in the disease course include progressive arthropathy, hip dysplasia, dysostosis multiplex, spine deformities, and spinal cord compression. Cardiac valve disease is also very common.

As shown in Table 1, the major clinical manifestations of attenuated MPS I are similar to those of MPS II, MPS VI, and MPS VII (Sly syndrome). MPS IV, Morquio syndrome, is characterized by a distinctive and severe skeletal dysplasia, dysplastic odontoid process, and hyperextendable joints/ligamentous laxity rather than joint contractures [5]. All of the MPS disorders occur across a spectrum of severity, and diagnosis is often challenging for those with the less severe forms of the disease.

### Joint Symptoms in MPS I

Joint symptoms in MPS I patients are almost universal, and in the case of attenuated disease are often the first symptom that brings a child to medical attention [1,2]. In the MPS I Registry, an ongoing, observational database that tracks natural history and outcomes of patients with MPS I, joint contractures are reported in over 80% of Hurler-Scheie and Scheie patients and usually precede diagnosis [14].

The basis for the abnormal development of bone and cartilage seen in the MPS disorders is not fully understood. Although the absence of clinical signs of inflammation is a hallmark of joint involvement in the MPS disorders, investigations conducted in animal models suggest paradoxically that the pathophysiology of bone disease involves inflammatory cytokines such as tumor necrosis factor α and interleukin-1β in addition to proteins important for lipopolysaccharide signaling (e.g., Toll-like receptor 4 and lipoprotein-binding protein) [15-17].

## Presentation of the Hypothesis

**Figure 1:**
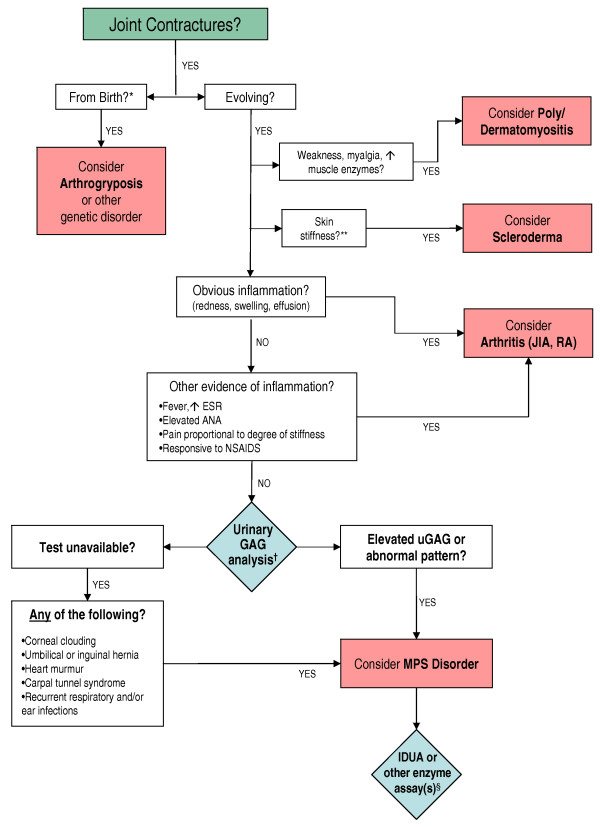
**Diagnostic Algorithm for Attenuated Mucopolysaccharidoses**. *Newborn infants with the most severe form of MPS I (Hurler syndrome), although normal appearing, often have radiologic evidence of bone and joint abnormalities. **Note that overall skin texture in patients with MPS I can be thickened and rough. MPS II [6,7] and rarely MPS I [30] can be associated with a distinctive skin lesion consisting of white "pebbly" papules 2-10 mm in diameter, sometimes coalescing in ridges. †We recommend both quantitative and qualitative (GAG profile) analysis in a reputable laboratory. False negatives can occur with spot screening. ^§^See Table 1 for listing of enzyme deficiencies. Abbreviations: IDUA: α-L-iduronidase; uGAG: urinary glycosaminoglycan; JIA: juvenile idiopathic arthritis; RA: rheumatoid arthritis.

**Figure 2:**
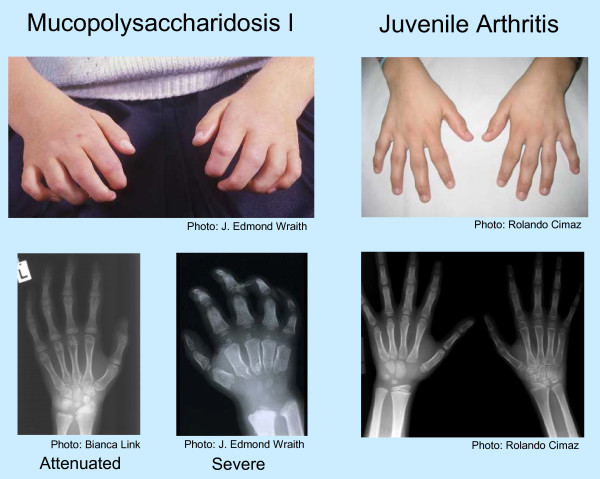
**Physical and Radiographic Appearance of Hands in MPS I versus Juvenile Idiopathic Arthritis**. The images on the left depict typical findings in MPS I - the curled "claw hand," abnormal metacarpal bones, proximal widening of phalanges, and the V-shaped deformity of the distal ulna and radius, particularly evident in severe MPS I. In contrast, the images on the right of a child with juvenile arthritis show the typical joint swelling and erosive bone lesions. Photos courtesy of J. Edmond Wraith, Rolando Cimaz, and Bianca Link.

**Figure 3:**
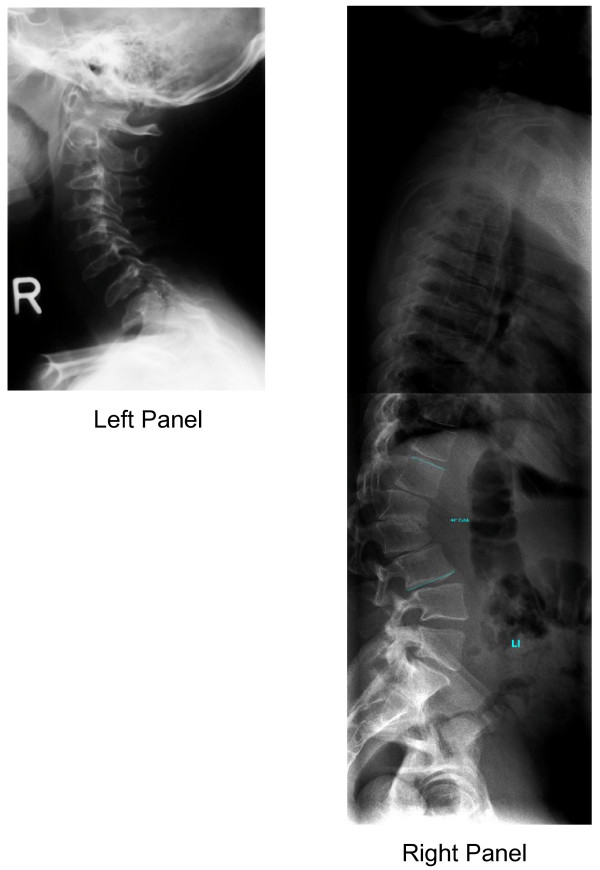
**Spine in Attenuated MPS I**. **Left Panel**: Cervical spine X-ray of an adolescent with attenuated MPS I showing ossification disturbance in the anterior part of column resulting in oval shaped vertebral bodies with anterior beaking and hypoplastic dens axis. Photo courtesy of Bianca Link. **Right Panel**: Complete spine X-ray in an adult patient with attenuated MPS I showing nearly normal shaped vertebral bodies and gibbus deformity at the thoracolumbar junction. Note that lateral X-rays of the thoracolumbar spine can also reveal anterior beaking. Photo courtesy of Bianca Link.

To aid rheumatologists and other physicians in the differential diagnosis of MPS I and other MPS disorders, a diagnostic algorithm is proposed (Figure 1), which was drafted at a one-day meeting attended by all authors. Joint contractures were chosen as a starting point in the algorithm because they are an early and almost universal symptom among MPS patients with attenuated disease. In summary, joint pain and joint contractures in the absence of systemic and local signs of inflammation should always raise the suspicion of an MPS disorder, especially in concert with any other common sign or symptom of MPS. Note that in cases of juvenile idiopathic arthritis characterized by "dry arthritis," joints usually lack synovial effusions, but patients may display other signs of an inflammatory process, such as frequent fevers and/or high erythrocyte sedimentation rate [18]. In contrast, MPS patients typically have no swelling or local inflammation of any joints, no morning stiffness, no laboratory indicators of inflammation (erythrocyte sedimentation rate, C-reactive protein, and white blood cell counts are not elevated), no response to steroids or non-steroidal anti-inflammatory drugs, and no radiographic evidence of erosive bone lesions (Figure 2 and 3). Indeed, the problems are persistent and often progress, particularly in patients not on enzyme therapy.

**Figure 4:**
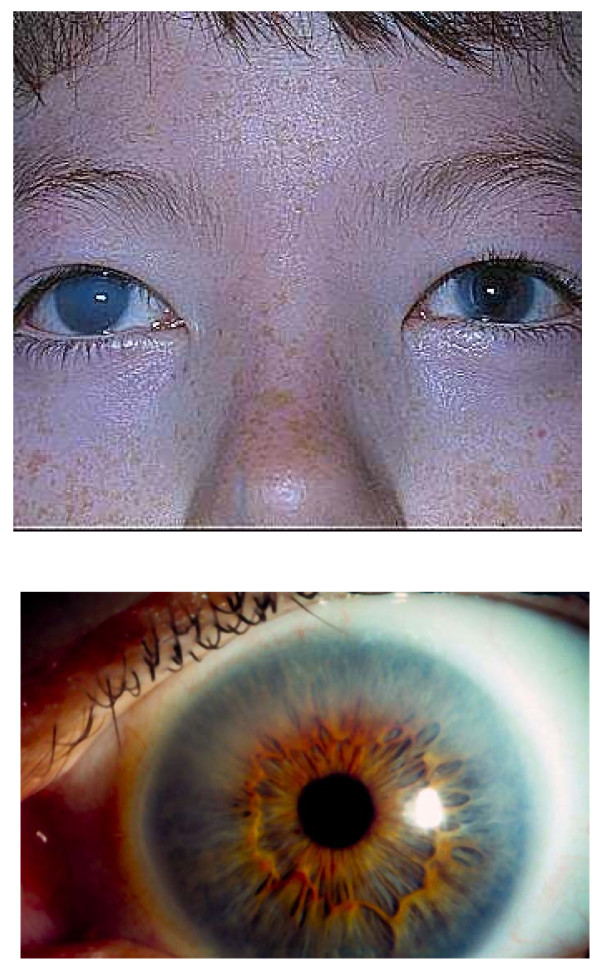
**Corneal Clouding in Patients with MPS I**. Corneal clouding is an early and almost universal sign of MPS I in both severe and attenuated phenotypes. It can be very marked, as in the right eye of the patient shown in the upper panel, or less obvious as in the eye shown in the lower panel. Typically, it is bilateral; in the patient shown in the upper panel, a corneal transplant was performed in the left eye. Photos courtesy of J. Edmond Wraith.

When MPS I or other MPS disorder is suspected, a urinary glycosaminoglycan (uGAG) analysis (both quantitative and qualitative) should be performed at a reputable laboratory. We do not recommend a spot screen, as they are inaccurate and false negatives can occur. An elevated uGAG level is a strong indicator of an MPS disorder; however, a normal uGAG level does not rule out all forms of MPS, some of which are best identified by an abnormal uGAG profile [6,7,19]. An elevated uGAG level and/or an abnormal uGAG pattern confirms the presence of an MPS disorder and specific enzyme testing will determine the MPS type. If comprehensive uGAG analysis is not available, enzymatic testing should still be performed if the patient exhibits any other common sign or symptom of an MPS disorder, such as corneal clouding (Figure 4) or history of hernia surgery, frequent respiratory and/or ear, nose and throat infections, carpal tunnel syndrome, or heart murmur. Table 1 lists the specific enzyme deficiency characteristic of each disorder. A definitive diagnosis of MPS I is based on deficient IDUA activity in fibroblasts, leukocytes, serum, or blood spots [20,21].

### Treatment

Patients with confirmed MPS should be referred to a geneticist or metabolic specialist for further evaluation and treatment. All of the MPS disorders are multisystemic and progressive and are best managed by a multidisciplinary team with comprehensive followup [5,10,22]. Both disease-specific treatment as well as symptom-based supportive care are important, in addition to patient education, genetic counseling, psychosocial support, and physical and/or occupational therapy. MPS patients can have significant (and often under-recognized) airway problems and cervical spine issues such as spinal cord compression that increase risk of anesthetic complications; thus, appropriate precautions should be taken before any surgery.

Disease-specific care for patients with attenuated MPS I consists of enzyme replacement therapy with laronidase (0.58 mg/100 U per kg per week). Available enzyme replacement therapies for the other MPS diseases with musculoskeletal manifestations are listed in Table 1. As with any progressive metabolic disease, early initiation of therapy that addresses the underlying pathophysiology improves outcome, and can prevent or at least delay the development of irreversible disease manifestations. Clinical trials of laronidase in patients with attenuated MPS I have demonstrated improved respiration, decreased hepatomegaly, increased walking ability, enhanced joint range of motion, and improved quality of life [23-26]. Hematopoietic stem cell transplantation (HSCT) is recommended for patients with severe MPS I, if performed before age 2 as it can prolong life and preserve cognition, but is not recommended for patients with attenuated MPS I, because they have little or no cognitive involvement, making the procedural risk-benefit ratio unacceptable (10-15% mortality) [10]. HSCT is used much less frequently in other MPS disorders and only among severely affected patients and transplant outcome has been mixed [7,27-29].

## Testing of Hypothesis

We propose that clinicians, particularly rheumatologists, pediatric rheumatologists, and orthopedists use our diagnostic algorithm when evaluating any patient with joint pain, stiffness or contractures and no systemic and local signs of inflammation. The index of suspicion for MPS should be especially high if the patient also has a history of hernia surgery, frequent respiratory infections, corneal clouding, carpal tunnel syndrome, or cardiac valvular abnormalities.

## Implications of Hypothesis

Attenuated MPS disorders can present a diagnostic challenge because the symptoms are often subtle and/or non-specific. Rheumatologists, and especially pediatric rheumatologists, are uniquely positioned to recognize many patients with attenuated MPS because of their characteristic joint involvement. Use of the proposed diagnostic algorithm should improve disease recognition, and enable more patients to receive appropriate treatment earlier in the course of their disease, maximizing clinical outcome and enhancing patient quality of life.

## Abbreviations

IDUA: α-L-iduronidase; HSCT: hematopoietic stem cell transplantation; MPS: mucopolysaccharidosis; uGAG: urinary glycosaminoglycan.

## Consent

Written informed consent was obtained from the patient for publication of the accompanying images. A copy of the written consent is available for review by the Editor-in-Chief of this journal.

## Competing interests

All authors received honoraria from Genzyme Corporation for attending the meeting at which the algorithm was drafted. In addition, RC has received reimbursement for travel expenses from Genzyme; GVC has received reimbursement for travel expenses and honoraria for presentations and advisory board meetings, BM has a consultancy agreement with Genzyme, GP is a recipient of research funding from Genzyme, and NW has received reimbursement for travel expenses and consultancy agreements from Genzyme. No other author has declared competing interests.

## Authors' contributions

An initial version of the algorithm was drafted by RC. BM chaired the meeting. All authors contributed equally to the development of the text, the revision of the algorithm, and the decision to submit to Pediatric Rheumatology. All authors have read and approved the final manuscript.

## Appendix

### Common Misdiagnoses for MPS I Based on Joint Symptoms [1,2,4]

• Autoimmune disease

• Muscular dystrophy

• Connective tissue disease

• Osteogenesis imperfecta

• Dermato/polymyositis

• Polymyositis

• Dermatomyositis

• Polyneuropathy

• Fibromyalgia

• Rheumatoid arthritis

• Growing pains

• Scleroderma

• Juvenile idiopathic arthritis

• Spondyloarthritis

• Legg-Perthes disease

• Other systemic rheumatic disorder
